# Differentially Expressed Genes in Clear Cell Renal Cell Carcinoma as a Potential Marker for Prognostic and Immune Signatures

**DOI:** 10.3389/fonc.2021.776824

**Published:** 2021-12-16

**Authors:** Ying Tong, Yiwen Yu, Hui Zheng, Yanchun Wang, Suhong Xie, Cuncun Chen, Renquan Lu, Lin Guo

**Affiliations:** ^1^ Department of Clinical Laboratory, Fudan University Shanghai Cancer Center, Shanghai, China; ^2^ Department of Oncology, Shanghai Medical College, Fudan University, Shanghai, China

**Keywords:** differentially expressed genes, hubs, immune infiltration, subgroups, immune checkpoint blockade, clear cell renal clear cell carcinoma

## Abstract

Clear cell renal cell carcinoma (ccRCC) is characterized by the inactivation of the von Hippel–Lindau (*VHL*) gene. Of note, no other gene is mutated as frequently as *VHL* in ccRCC, turning out that patients with inactivated *VHL* constitute the majority of ccRCC-related character. Thus, differentially expressed genes (DEGs) and their molecular networks caused by *VHL* mutation were considered as important factors for influencing the prognosis of ccRCC. Here, we first screened out six DEGs (*GSTA1*, *GSTA2*, *NAT8*, *FABP7*, *SLC17A3*, and *SLC17A4*) which downregulated in ccRCC patients with *VHL* non-mutation than with the mutation. Generally, most DEGs with high expression were associated with a favorable prognosis and low-risk score. Meanwhile, we spotted transcription factors and their kinases as hubs of DEGs. Finally, we clustered ccRCC patients into three subgroups according to the expression of hub proteins, and analyzed these subgroups with clinical profile, outcome, immune infiltration, and potential Immune checkpoint blockade (ICB) response. Herein, DEGs might be a promising biomarker panel for immunotherapy and prognosis in ccRCC. Moreover, the ccRCC subtype associated with high expression of hubs fit better for ICB therapy.

## Introduction

Renal cell carcinoma (RCC) has become a common but deadly genitourinary malignancy with an increasing incidence, with an estimate of 73,750 new cases and 14,830 death cases in the US alone (1). Of note, due to its being symptomless in the early stage and poorer prognosis, the clear cell type has already taken up to approximately 80% of all RCC subtypes, constituting the majority of cancer-related deaths (2). In the early stage, clear cell renal cell carcinoma (ccRCC) patients can be treated with surgical or ablative strategies with a great outcome, whereas, metastases will still happen in approximately 30% of ccRCC patients (3) with a high mortality rate in advanced phases due to poor responses to radiotherapy and chemotherapy (4). It is reported that the incidence of somatic von Hippel–Lindau (*VHL*) mutations in sporadic ccRCC occupies up to 91% (5). Understanding differentially expressed genes (DEGs) and their molecular networks caused by *VHL* mutation has been and will continue to be critical to the development to improve both treatment and management of ccRCC patients.

It is known to all that a part of patients has pronounced clinical response with therapeutic intervention; however, other patients still gained minimal or no clinical benefit when provided the same treatment in the same tumor type. More literature uncovered the complexity and diversity of the immune context of the tumor microenvironment (TME) and its influence on response to therapy (6, 7). TME formed by many distinct and interacting cell populations, response, and survival benefit is typically limited to a subset of patients. Thus, patients should be divided into subgroups to investigate separately in clinical research studies. Based on the putative role of TME in influencing the prognosis, immune infiltration analysis has attracted increasing attention in recent years. Identification and comprehensive characterization subtypes are needed for designing novel immunotherapies so as to better improve the response and outcomes of ccRCC patients. Immune checkpoint blockade (ICB) can elicit durable clinical responses by reactivating an exhausted immune response and unprecedented clinical benefit in a subset of patients across multiple types of solid tumors (8–10). For patients with a favorable immune microenvironment, ICB can be used to enhance the preexisting antitumor immunity of these patients and further improve their survival (11).

Seeking factors that specifically influence the prognosis in ccRCC is critical to improving the treatment and management of ccRCC patients. Investigation and characterization of DEGs are vital for seeking prognosis influencing factors. Thus, we first screened out six DEGs between ccRCC patients with *VHL* mutation and non-mutation. Proverbially, a broad network of molecular changes is involved in influencing and modulating DEGs. Thereupon, we analyzed the upstream regulating kinases and transcription factors (TFs) of DEGs by a systems biology approach and defined them as hub proteins. Moreover, the complexity and diversity of TME signify that only a part of patients benefits from therapeutic intervention. So, we clustered ccRCC patients into three subgroups according to their expression of hub proteins, and whereafter analyzed with clinical profile, outcomes and immune infiltration. In general, our results demonstrated that DEGs might be supposed as promising prognostic biomarkers in ccRCC and might have clinical implications for personalized immunotherapy.

## Materials and Methods

### ccRCC Datasets

Protein expression of 84 adjacent normal tissues and 110 ccRCC samples were derived from the Clinical Proteomic Tumor Analysis Consortium (CPTAC). Raw counts of RNA-sequencing data (level 3) of patients with ccRCC (n = 530) and the corresponding clinical data were downloaded from TCGA data portal, in which the method of acquisition and application complied with the guidelines and policies. Data on overall survival (OS) were extracted from TCGA cohort.

### Differential Genes Expression Analysis of ccRCC

The raw count data of mRNA profile in ccRCC from TCGA dataset include both *VHL* wild-type and mutation groups. Volcano plots were used to filtrate the DEGs *via* Limma package (version: 3.40.2) of R software. DEGs was constructed using fold-change values and adjusted P. The adjusted P-value was analyzed to correct for false positive results in TCGA or GTEx. Adjusted P <0.05 and Log (Fold Change) >1 or Log (Fold Change) <−1 were charactered as the thresholds for the screening of DEGs.

### Protein Expression Analysis

We explored the total protein expression level of DEGs between primary tumor and normal tissues through the *CPTAC* (Clinical proteomic tumor analysis consortium) dataset in the *UALCAN* portal (http://ualcan.path.uab.edu/analysis-prot.html), an interactive web resource for analyzing cancer Omics data. The available dataset of clear cell RCC was selected.

### Functional Enrichment Analysis of DEGs

With the screened DEGs, Kyoto Encyclopedia of Genes and Genomes (KEGG) pathways analysis and gene ontology (GO) enrichment analysis were performed on the online tool *Metascape* (http://metascape.org/gp/index.html#/main/step1).

### Spearman Correlation Analysis

The dataset was comprised of mRNA-seq data of ccRCC cohort from TCGA. Spearman’s correlation analysis was used to describe the correlation among DEGs without a normal distribution. The multi-gene correlation map was realized by the R software package pheatmap. The value in the sphere represented the correlation p-value and the bigger sphere represents the stronger correlation.

### Infer Upstream Regulatory Networks

Upstream regulatory networks from signatures of DEGs were inferred by X2K Web. Transcription Factor Enrichment Analysis (TFEA) is the first step of the X2K pipeline, which was predicted to regulate DEGs by performing gene set enrichment analysis using ChIP-seq experiments (ChEA). Secondly, Protein–Protein Interaction (PPI) Expansion was the expansion of enriched TFs, achieved by identifying proteins which physically interact with these TFs through the Genes2Networks (G2N) algorithm. Finally, Kinase Enrichment Analysis (KEA) was the third step of the X2K pipeline, which performed enrichment analysis based on the list of proteins from the PPI network using kinase–substrate interaction databases.

### Prognostic Risk Signatures

The prognostic risk signatures of DEGs were established by the least absolute shrinkage and selection operator (LASSO) regression analysis in the TCGA set. Coefficients of selected features were shown by λ parameter. Signatures were screened out by selecting the optimal penalty parameter λ correlated with 10-fold cross-validation using the R software package glmnet. Partial likelihood deviance versus log (λ) was drawn using LASSO Cox regression model. Kaplan–Meier survival analysis with log-rank test was also used to compare the survival difference between low-risk and high-risk group. The analytical methods were performed by R software. The hazards ratio was calculated based on Cox PH Model.

### Immune Score and Immune Infiltration Analysis

In the “Immune-Gene” module of the TIMER2 web, we explored the association between the expressions of hub genes and immune infiltrates across in the ccRCC. To make reliable immune infiltration estimations and immune cell distribution score, we took advantage of the immunedeconv, an R package which integrates some state-of-the-art algorithms, namely, MCPCOUNTER and EPIC. The p-values and the correlation (cor) values in ccRCC were achieved *via* the purity-adjusted Spearman’s rank correlation test. The data were visualized as a heatmap and a scatter plot.

### Characterization of ccRCC Subgroups and Immune Signature Analysis in Subgroups

Raw counts of RNA-sequencing data and corresponding clinical information of ccRCC were obtained from TCGA dataset. ConsensusClusterPlus (v1.54.0) for consistency analysis was implemented by R software package. Clustering heatmaps were conducted *via* R software package pheatmap. The gene expression heatmap retains genes with SD >0.1.

### Immune Checkpoint Analysis

CD274, CTLA4, HAVCR2, LAG3, PDCD1, PDCDILG2, TIGIT, and SIGLEC15 were genes relevant to immune-checkpoint, and expression values of these eight genes were extracted. Immune checkpoints related gene expression were implemented by packages ggplot2 of R foundation for statistical computing (version 4.0.3). The significance of the three groups has passed the Kruskal–Wallis test.

### Predict Potential ICB Response

We collected the RNA-sequencing data and corresponding clinical information of ccRCC from TCGA dataset. Potential ICB response was predicted with TIDE algorithm (12).

## Results

### Analysis of Differentially Expressed Genes in ccRCC

**Figure 1:**
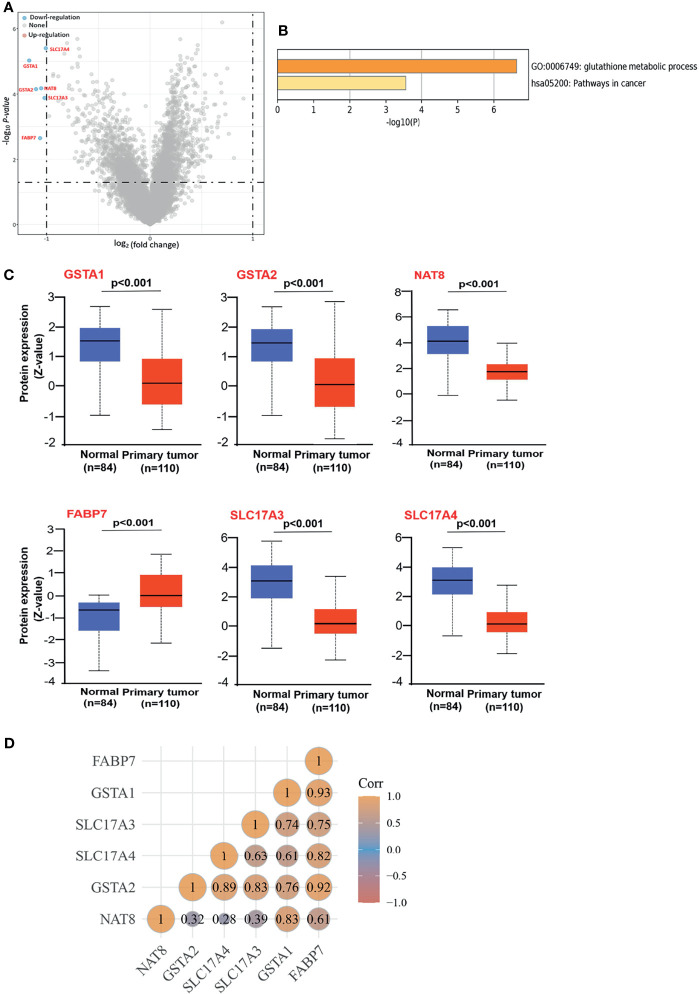
Screening out the DEGs and identifying their characteristics, **(A)** Volcano plots to screen out the DEGs between patients with wild-type and mutation VHL. The blue point indicates the down-expressed mRNAs with statistical significance. The default set of the threshold was fold change ≥2, P ≤0.05. **(B)** GO Enrichment analyses of DEGs. GO, gene ontology. **(C)** Based on the CPTAC dataset, we analyzed the total protein expression level of GSTA1, GSTA2, NAT8, FABP7, SLC17A4, and SLC17A4 in normal tissue and primary tissue of ccRCC. **(D)** Spearman correlation analysis between DEGs. The value represents the correlation p-value, and the darker the color represents a stronger correlation.

Since the inactivation of the *VHL* is the signature initiating event in ccRCC, we examined the DEGs between ccRCC patients with *VHL* mutation and non-mutation using Volcano plots. Six genes (*GSTA1*, *GSTA2*, *NAT8*, *SLC17A3*, *SLC17A4*, and *FABP7*) were screened out, downregulating in ccRCC patients with wild-type *VHL* compared to patients with the mutation (**Figure 1A**). Enrichment analyses using GO were performed to investigate the potential roles of DEGs, indicating that DEGs were involved in both the processes of glutathione metabolic and the pathways in cancer (**Figure 1B**). To better explore the roles of DEGs in ccRCC, we evaluated the protein expressions of DEGs in cancerous and normal tissues. As the *CPTAC* dataset exhibited, the total protein expression levels of GSTA1, GSTA2, NAT8, SLC17A3, and SLC17A4 were much lower in cancerous than those in normal tissues (**Figure 1C**). Meanwhile, to probe the consistency of DEGs in ccRCC, we performed Spearman correlation analysis in TCGA database. As the average Pearson correlation emerged above, DEGs tended to be positively correlated with each other (**Figure 1D**). Above all, DEGs were involved in the pathway of carcinogenesis with a low expression in ccRCC on the whole.

### Prognostic Analysis of DEGs Signature in ccRCC

**Figure 2:**
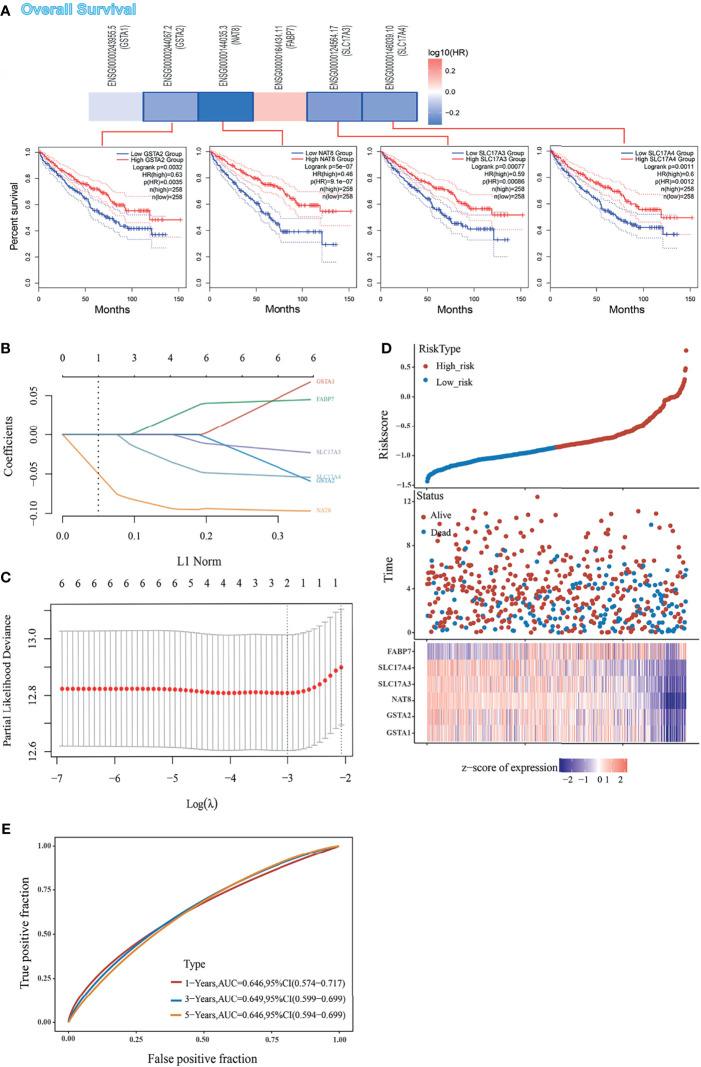
Prognostic value of DEGs in ccRCC cohort form TCGA set, **(A)** The correlation between OS and DEGs expression, and Kaplan–Meier curves performed by GEPIA2 tool describes the OS. **(B)** Coefficients of DEGs are shown by lambda parameter. **(C)** Partial likelihood deviance versus log (λ) was drawn using LASSO Cox regression model. **(D)** Patients were divided into low- and high-risk groups according to risk score (upper). Survival status of the patients (middle) and expression profiles of the DEGs (below) are shown corresponding to the risk score. **(E)** Time-dependent ROC analysis of the DEGs. The AUC value predicts the prediction ability of the model.

Although enrichment analyses have provided a potential forecast that DEGs may participate in the cancer pathway, our knowledge of DEGs was still limited to its expression difference. On this basis, we envisaged to analyze the prognostic signature of DEGs in the TCGA set. Firstly, we divided the ccRCC patients into high-expression and low-expression groups according to the expression levels of DEGs and further investigated the correlation between DEGs expression and the prognosis of patients, mainly using the datasets of TCGA. Analysis from *GEPIA2* indicated that the high expressions of *GSTA2*, *NAT8*, *SLC17A3*, and *SLC17A4* were linked to favorable prognosis of OS (**Figure 2A**). LASSO coefficients of DEGs were shown by lambda parameter (λ) (**Figure 2B**). LASSO Cox regression model was performed to get the optimal lambda value that came from the minimum partial likelihood deviance (λmin  =  0.0015), which was related with DEGs that significantly associated with OS (**Figure 2C**). Furthermore, DEGs-based risk score was constructed based on their Cox coefficients: Riskscore = (−0.0636) ∗ GSTA1+ (−0.0558) ∗ GSTA2+ (−0.0968) ∗ NAT8 + (−0.0223) ∗ SLC17A3 + (−0.054) ∗ SLC17A4 + (0.0447) ∗ FABP7. The dotted line represented the median risk score and divided the patients into low-risk and high-risk group. The survival status of all patients was shown in the training group, while a heatmap presented the expression profiles of DEGs in both low-risk and high-risk group (**Figure 2D**). Finally, the prognostic signature of DEGs showed that larger AUC values in a time-dependent ROC analysis was positively related to a better predictive ability of multi-gene model for 1-, 3-, and 5-year OS (**Figure 2E**). To sum up, high expressions of *GSTA2*, *NAT8*, *SLC17A3*, and *SLC17A4* forebode a favorable prognosis, while the lower expression of all DEGs except *FABP7* might predict a higher risk of ccRCC.

### Spot the Major Hub Proteins of the DEGs

**Figure 3:**
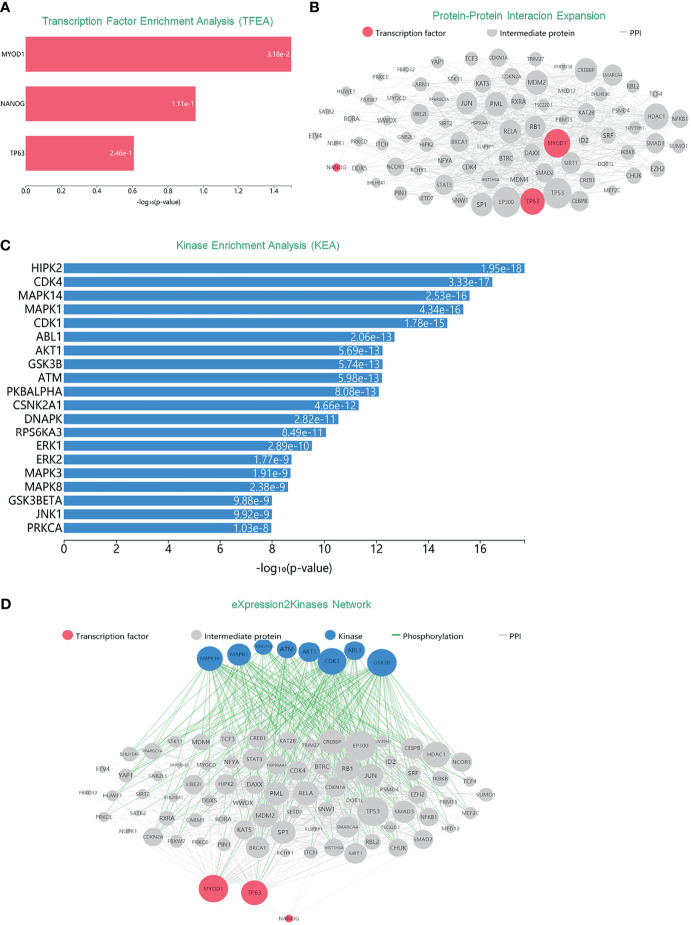
Inferred upstream regulatory networks of DEGs. **(A)** TFEA was used to predict TFs. Top predicted TFs (MYOD1, NANOG, and TP63) are displayed as a bar graph. **(B)** PPI expands the list of enriched TFs. Gray edges indicate the interaction between two proteins. The size of nodes is relative to the level of expression degree. **(C)** KEA predicts the protein kinases that are likely the regulators of the expanded PPI network. **(D)** The eXpression2Kinases network displays the inferred upstream regulatory network of the DEGs.

Many human cancers are dependent on the inappropriate activity of oncogenic TFs (13), turning out that TFs were well-connected nodes which had a strong capacity of modulating adjacent genes. In order to extensively investigate DEGs mentioned above, and also their role within signaling networks or their transcriptional regulatory mechanisms, we conducted TFEA, screening out three TFs of DEGs with high predictive value, which were MYOD1, NANOG and TP63 as **Figure 3A** manifested. Moreover, results of Protein–Protein Interaction (PPI) networks established a basic abstraction of multiple complex pathways, controlling the major cellular and molecular machinery which could determine the disease. By identifying proteins that physically interact with MYOD1, NANOG, and TP63, a subnetwork of connected TFs and their interacting proteins was visualized as a ball-and-stick diagram (**Figure 3B**). Since protein kinases are responsible for cellular transduction signaling and their hyperactivity, we conducted a Kinase Enrichment Analysis (KEA), and filtered the top predicted kinases that probably regulated the expanded PPI network (**Figure 3C**). Furthermore, the eXpression2Kinases (X2K) network was performed to infer upstream regulatory network (**Figure 3D**). Generally, MYOD1, NANOG, and TP63, along with 20 kinases mentioned in **Figure 3C**, were all supposed to be hub proteins of the DEGs. Phosphorylation orchestrates the activity and stability of hub genes, where alterations in phosphorylation pathways were of great importance to the networks mentioned above.

### Analysis of Hub Proteins Identifies Distinct Subgroups of ccRCC

**Figure 4:**
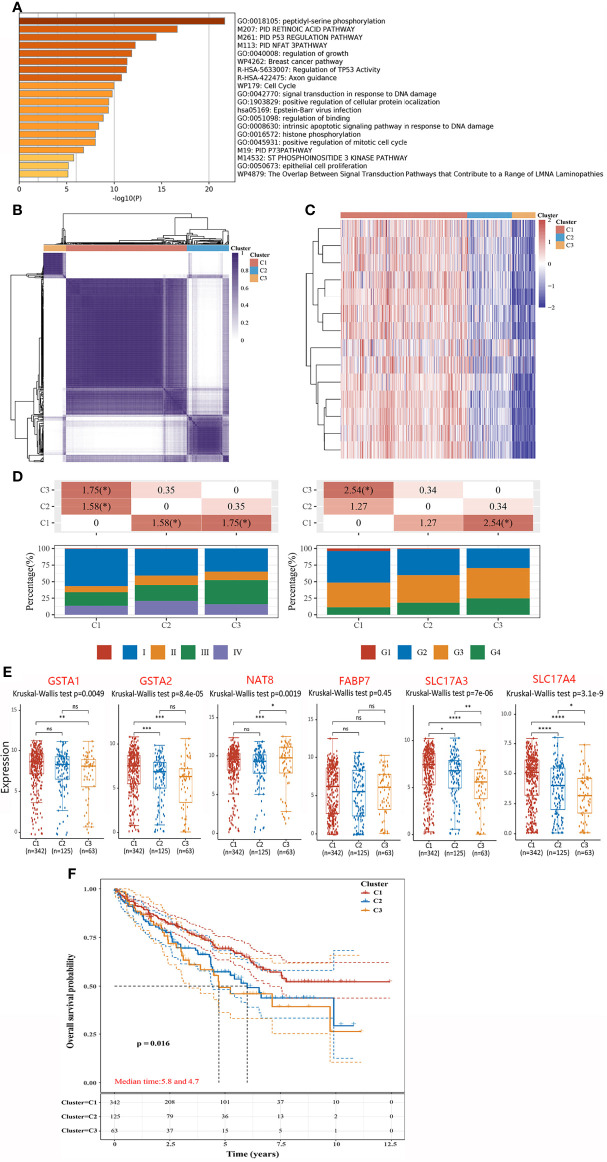
Integrated cluster assignments analysis of ccRCC patients and characteristics described of them. **(A)** GO Enrichment analyses based on hub genes. **(B)** Heatmap depicting consensus clustering solution (clusters = 3). **(C)** Heatmaps showing the expression of hubs in three subgroups. Red represents high expression and blue represents low expression. **(D)** Distributions of tumor stage (left) and the degree of progression of the primary tumor (right) in three subgroups. The table above represents the distribution of a certain clinical feature in any two subgroups. The significance p-value was analyzed by chi-square test, where the value is −log10 (p value), if marked with *, it means that there is a significant difference in the distribution of the clinical feature in the corresponding two groups (p <0.05). **(E)** The expressions of DEGs in three subgroups. **(F)** Kaplan–Meier curves describe the OS for three subgroups. *P < 0.05, **P < 0.01,***P < 0.001, ns, no significance.

PTMs are involved in varieties of cellular activities, and phosphorylation is one of the most extensively studied PTM (14). To further investigate the molecular mechanism of hub genes, we performed KEGG and GO enrichment analyses. Results indicated that most of these genes were linked to orchestrating a range of intracellular processes including cell growth, proliferation, division and so on (**Figure 4A**). In recent years, the concept of precision medicine has promoted the subgroups of individual research objects, and different subgroups have different pathogenic mechanisms and clinical prognostic characteristics. To achieve a general and overall evaluation of hub genes among ccRCC patients, we classified the cohort (n = 530) into three different subtypes *via* consistency analysis according to the expression level of hub genes (**Figure 4B**). Remarkably, all hub genes were considerably high expressed in C1 subgroup while low expressed in C3 (**Figure 4C**). To investigate the clinical profile of three subtypes, tumor stage and the degree of progression of the primary tumor were compared among patients in three subtypes. Clinically, cluster C1 presented a strikingly higher frequency of Stage I and early grade (G1 + G2), and a lower frequency of advanced grade (G3 + G4) than the other two subtypes did (**Figure 4D**). Likewise, expression levels of DEGs except *FABP7* were higher in C1 than those in C3 (**Figure 4E**). Upon stratification of the three clusters according to specific data sets, significant differences in OS were observed between the three subtypes. Notably, subtype C1 had the highest OS rate among the three clusters. In comparison, patients with subtype C3 had a worse OS than the other two (p = 0.016) (**Figure 4F**). In general, hub genes were serviceable for delineating ccRCC clusters, and the DEGs have the prognostic values in clustered ccRCC subtypes. Namely, the subtype with higher expressions of DEGs might be associated with better OS.

### Immune Infiltration Analysis of ccRCC Subtypes

**Figure 5:**
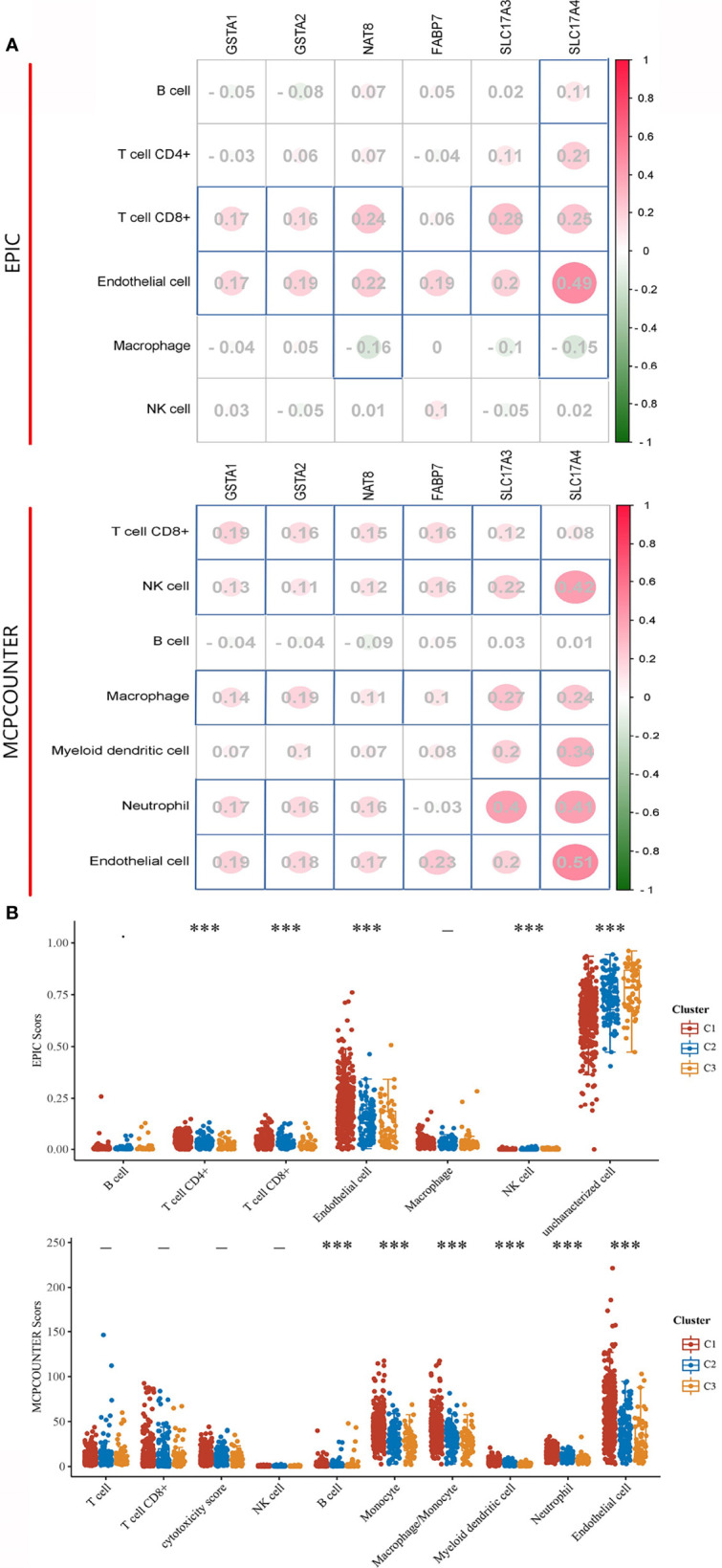
Immune signature of DEGs and three subgroups in ccRCC. **(A)** The correlations between DEG and immune cells. The corresponding P-values were shown in parenthesis. The left panel shows varieties of immune cells and algorithms. **(B)** The abundance score of infiltrating immune cells across each subgroup. The significance of the three groups passed by the Kruskal–Wallis test. ***P < 0.001, -: no significance.

Inflammation and immune evasion are considered as hallmarks of cancer progression, highlighting the direct involvement of immune cells (15). As the prognostic value of DEGS for ccRCC patients subjected to OS has already been estimated, we explored whether the DEGs could influence the immune infiltration in ccRCC patients. The relationship between the expression distribution of DEGs and infiltration levels of immune cell types was analyzed to estimate the effect of DEGs on the immune microenvironment. In terms of EPIC algorithms, a significantly positive correlation was observed between most of the DEGs and infiltration levels of T cell CD8+ and endothelial cell. While analyzed by MCPCOUNTER algorithms, most of the DEGs positively correlated to T cell CD8+, NK cell, Macrophage, Neutrophil, and endothelial cell (**Figure 5A**). Given the heterogeneity of TME across ccRCC patients (16), it is quite likely that the immune cells might also vary across these subgroups. Thus, we calculated several types of immune cells between three subgroups by EPIC and MCPCOUNTER algorithms. According to EPIC algorithms, a higher number of immune-associated cells such as, CD4+, CD8 T cells, and endothelial cell were produced in subtype C1 than in other subtypes. Additionally, assessed with MCPCOUNTER algorithms, B cell, monocyte, macrophage, myeloid dendritic cell, neutrophil, and endothelial cell were more aggressively to subtype C1 (**Figure 5B**). It was noted that DEGs were positively related to immune cell score and a higher number of immune-associated cells distributed in subtype C1, showing higher expression of DEGs contribute an enhanced immune microenvironment in C1.

### Immune Checkpoint and Predicts ICB Response

**Figure 6:**
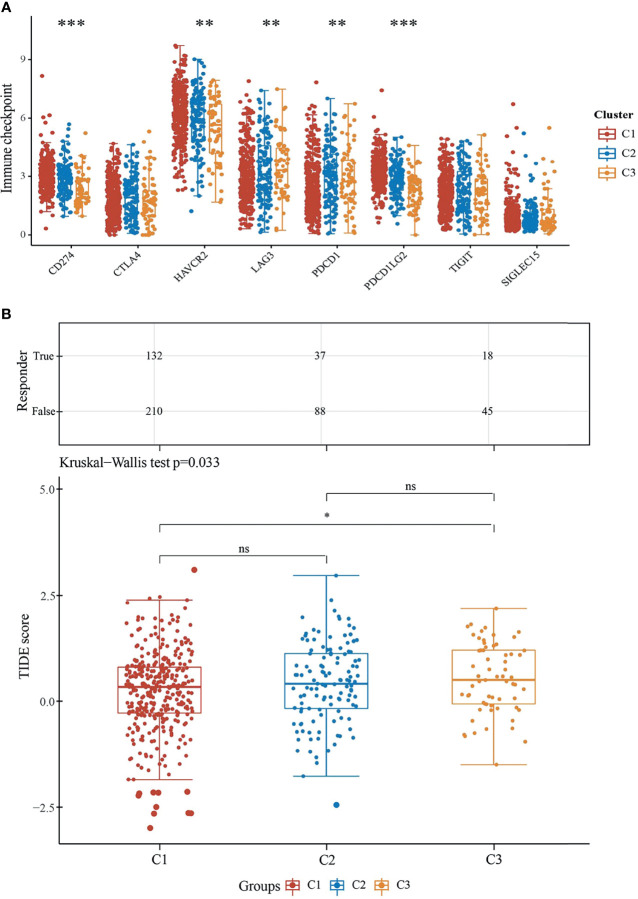
Predictive ICB response of ccRCC subgroups. **(A)** Expressions of eight immune checkpoint molecules in three subgroups. The significance of the three groups and above passed the Kruskal–Wallis test. **(B)** Statistical table of immune response samples (upper) and the distribution of immune response scores (underneath) in ccRCC subgroups in prediction. **P < 0.01, ***P < 0.001, ns, no significance.

Immune checkpoint molecules are ligand–receptor pairs that exert inhibitory or stimulatory effects on immune responses (17). Most of them have been deemed to express on cells of the adaptive immune system, particularly on T cells, and also innate immune system (18). We explored the expression values of immune checkpoint molecules (SIGLEC15, TIGIT, CD274, HAVCR2, PDCD1, CTLA4, LAG3, and PDCD1LG2). Indeed, the expressions of CD274, HAVCR2, and PDCD1LG2 were unanimously higher in C1 than the other two subtypes (**Figure 6A**) and were in accordance with the infiltration levels of principally immune cells assessed in previous sections. Moreover, cancer immunotherapies by ICB aimed to assist the immune system to recognize and attack cancer cells (19). We next evaluated ICB clinical response by Tumor Immune Dysfunction and Exclusion (TIDE), a computational method to model two primary mechanisms of tumor immune evasion (12). As reported, a higher TIDE score forebodes a poorer efficacy of ICB therapy and less favorable survival after ICB treatment. Excitingly, the immune response samples were higher in C1 than in C3. On the contrary, the TIDE score was significantly lower in C1 than in C3 (**Figure 6B**). According to our results, subtype C1 fits better for ICB therapy.

## Discussion

Over 400,000 individuals are affected by renal cell carcinoma worldwide per year, while approximately 70% are diagnosed as ccRCC. Although surgical resection, treated as a primary therapy for localized tumors, can achieve a great outcome, there still exist 30% of ccRCC patients suffering from metastases, with a 5-year survival rate of 8–12% (20). Since the von Hippel–Lindau (*VHL*) gene has been identified in 1993, the *VHL* mutations have been reported as one of the genetic determinants which promote the initiation and progression of ccRCC (5, 21, 22). After years of exploring different prognostic markers in ccRCC, it still remains a challenge for us to find a stable and reliable biomarker to predict the OS. In our study, we used volcano plots to identify the DEGs between ccRCC patients with wild-type *VHL* and *VHL* mutation. Six genes (*GSTA1*, *GSTA2*, *NAT8*, *SLC17A3*, *SLC17A4* and *FABP7*) were screened out, given their downregulation in ccRCC patients with wild-type *VHL*. Furthermore, GO enrichment analysis illustrated that DEGs mentioned above were all involved in the pathway of cancer. Moreover, a prognostic analysis on DEGs and surprisingly found that *GSTA2*, *NAT8*, *SLC17A3*, and *SLC17A4* turned out to be inversely related to OS. Additionally, apart from *FABP7*, all DEGs could function as risk associated genes, while a lower expression might be related with a higher risk of ccRCC patients.

During the past few years, numerous studies have validated the role of multiple TF targets in cancer. Of note, the substantial potential of TFs to modulate several pathways of both cancer and other diseases has already drawn great attention (23, 24). In the present study, MYOD1, NANOG, and TP63 were the TFs that modulated DEGs. Moreover, the construction of a PPIs network provided insights for a basic abstraction of larger complex pathways which controlled the major cellular and molecular machinery determining the disease (25, 26). Here, a subnetwork based on the three TFs visualized the correlation among TFs and proteins interacted with them. Furthermore, results from KEA suggested that protein kinases might regulate the expanded PPI network. On the basis of expanded PPI network and KEA, we deemed that TFs and kinases had the capability of modulate adjacent genes. Because of their high connectivity, these proteins are called hub proteins (or hubs) and they are of critical importance to PPI networks and whole biological systems (27, 28). Thus, MYOD1, NANOG, and TP63, along with 20 kinases mentioned in **Figure 3C**, were all supposed to be hub proteins of the DEGs.

During the recent years, the term “precision medicine” has come into our sight and has become more and more popular both in scientific and political perspectives (29). Consequently, stratified medicine and tailored therapy have been scientifically developed so as to promote the clinical application (30). To achieve a general and overall evaluation of hub genes among ccRCC patients, we classified the cohort (n = 530) into three different subtypes *via* consistency analysis according to the expression level of hub genes. Meanwhile, the expression of DEGs, clinical characterization, and prognosis in three subtypes were analyzed as well. Surprisingly, we found that each subtype was associated with distinct clinical characteristics and DEGs expression profiles, contributing to different outcomes in OS.

There is a large volume of published studies describing the role of immune cells in host defense against both cancer and infection (15). Meanwhile, the immune-related signature has already been observed in such cancers as pancreatic cancer, glioblastoma, and most importantly, renal carcinoma (31–33), with plenty of specialized cell types involved in (34). It is known that *VHL* mutations can be widely detected in ccRCC; moreover, it has been suggested that *VHL* mutations might drive the activation of effector T cells as well as enhance cytokine level in ccRCC (35), indicating a potential but crucial role that immune infiltration might play. Although a large body of evidence points to the extent and functional orientation of the T cell infiltrate as important in therapy response (36, 37), recent studies also confirm a role for other components of the TME, such as B cells (38), myeloid lineage cells (39), NK cells (40), and macrophage (41). As the prognostic value of DEGs for ccRCC patients has been evaluated in our study, we aimed to get a better knowledge of the nature and differences of immune infiltration in ccRCC, especially in three subgroups. The correlations between DEGs and infiltrating immune cells, including B cells, CD4+T cells, CD8+T cells, endothelial cells, macrophages, and nature killer (NK) cells, were analyzed by EPIC and MCPCOUNTER algorithms. Considering the heterogeneity of TME among three subgroups of ccRCC classified above, we deeply investigated whether there existed differences among C1 to C3. Fortunately, results from both EPIC and MCPCOUNTER algorithms illustrated a higher number of immune-related cells in C1 than in other subgroups, implying a positive relation between DEGs and immune cell score, which might become a promising predictive panel for immunotherapy. Furthermore, the composition of the TME has been shown to influence response to ICB (42). Recent breakthroughs uncovered that ICB could take advantages of immune cell infiltration in tumors to reinvigorate an efficacious antitumoral immune response (42, 43). Our study confirmed that cluster C1 was supposed to have a higher efficacy of ICB therapy which was associated with a higher expression of immune checkpoint molecules and immune cell infiltration. According to our results, subtype C1 fits better for ICB therapy, suggesting a potential role of DEGs and hubs as a promising predictive strategy for ICB therapy response. Further studies will be performed to further investigate differences in immune microenvironment and ICB therapy response between patients with and without *VHL* mutation.

In conclusion, our study aimed to promote the evaluation of ccRCC prognosis and filtered out six DEGs which could be recognized as a promising prognostic panel for ccRCC and also a potential indicator for immunotherapy. Moreover, hub genes were identified in this study in classified ccRCC patients into three subgroups, proposing to provide a more specific evaluation of the prognosis, respectively and accurately. Furthermore, the ccRCC subtype associated with high expression of hubs fits better for ICB therapy, providing potential therapeutic implications for rational immunotherapy strategies.

## Data Availability Statement

The original contributions presented in the study are included in the article/**Supplementary Material**. Further inquiries can be directed to the corresponding authors.

## Author Contributions

Conceptualization, RL and LG. Formal analysis, YT. Writing—original draft, YY. Visualization, HZ. Methodology, YW. Software, SX and CC. All authors contributed to the article and approved the submitted version.

## Funding

This study was supported by the National Natural Science Foundation of China (Nos. NSF-81772774, NSF-81772808, and NSF-81800190), the National Science and Technology Major Project (2020ZX09201-013), and the Science and Technology Commission of Shanghai Municipality (19ZR1410300).

## Conflict of Interest

The authors declare that the research was conducted in the absence of any commercial or financial relationships that could be construed as a potential conflict of interest.

## Publisher’s Note

All claims expressed in this article are solely those of the authors and do not necessarily represent those of their affiliated organizations, or those of the publisher, the editors and the reviewers. Any product that may be evaluated in this article, or claim that may be made by its manufacturer, is not guaranteed or endorsed by the publisher.
